# A qualitative exploration of Malaysian cancer patients' perspectives on cancer and its treatment

**DOI:** 10.1186/1471-2458-11-525

**Published:** 2011-07-01

**Authors:** Maryam Farooqui, Mohamed A Hassali, Aishah K Shatar, Asrul A Shafie, Tan B Seang, Muhammad A Farooqui

**Affiliations:** 1 Faculty of Pharmacy, Universiti Teknologi MARA (UiTM), Pulau Pinang, Malaysia; 2 Discipline of Social & Administrative Pharmacy, Universiti Sains Malaysia (USM), Pulau Pinang, Malaysia; 3 Advanced Medical and Dental Institute, Universiti Sains Malaysia (USM), Pulau Pinang, Malaysia; 4 Department of Radiotherapy and Oncology, General Hospital Penang, Pulau Pinang, Malaysia; 5 Department of Medicine, Allianze University College of Medical Sciences (AUCMS), Pulau Pinang, Malaysia

## Abstract

**Background:**

Cancer patients' knowledge about cancer and experiences with its treatment play an important role in long-term adherence in their disease management. This study aimed to explore cancer patients' knowledge about cancer, their perceptions of conventional therapies and the factors that contribute to medication adherence in the Malaysian population.

**Methods:**

A qualitative research approach was adopted to gain a better understanding of the current perceptions and knowledge held by cancer patients. Twenty patients were interviewed using a semi-structured interview guide. A saturation point was reached after the 18^th ^interview, and no new information emerged with the subsequent 2 interviews. All interviews were transcribed verbatim and analysed by means of a standard content analysis framework.

**Results:**

The majority of patients related the cause of their cancer to be God's will. Participants perceived conventional therapies as effective due to their scientific methods of preparations. A fear of side effects was main reasons given for delay in seeking treatment; however, perceptions were reported to change after receiving treatment when effective management to reduce the risk of side effects had been experienced.

**Conclusions:**

This study provides basic information about cancer patients' perceptions towards cancer and its treatment. These findings can help in the design of educational programs to enhance awareness and acceptances of cancer screening. Priorities for future research should focus on patients who refused the conventional therapies at any stage.

## Background

Cancer is a leading cause of mortality and morbidity worldwide. The World Health Organization (WHO) estimated 7.6 million deaths due to cancer in the year 2005, a number expected to rise to 12 million deaths by the year 2030 [1]. Until 2001, Malaysia did not have a National Cancer Registry (NCR) to estimate the real incidence of cancer among Malaysians [2]. Evaluation of NCR reports published from 2002-2006 indicates that the number of new cancer cases among residents in Peninsular Malaysia has decreased from 26,089 to 21,773 [3,4]. However, after taking into account unregistered cases, the probability that a Malaysian will get cancer in his/her life time is 1 in 4 [3]. Despite the decline in new cases, presentations at an advanced stage are still on the rise [5]. Lifestyle changes such as lack of regular exercise, consumption of a high fat diet, and breast feeding habits are reported as some important risk factors among Malaysian breast cancer patients [6].

Attitude towards cancer and its causes and treatment options are highly dependent on peoples' social and cultural background [7]. Numerous studies have been conducted globally to evaluate the meaning of cancer to patients from different ethnic groups [8,9]. Of particular note, a few indigenous minority groups lack a well-defined word for cancer in their native language [10]. Cancer is considered an incurable disease that is related to magic, bad luck, or a punishment from God, and more or less the same scenario is observed in developing countries [11,12]. Social and cultural perception, poor access to health care facilities and strong beliefs in alternative therapies are a few of the reasons for advanced stage presentation [11,13,14]. In a recently published Malaysian study, it was reported that breast cancer patients seek treatment from 2-36 months after the identification of a breast lump. Additionally, the study observed that 60% of the females used alternative therapies for cancer treatment [15]. Despite numerous efforts by the government to improve cancer awareness, poor response to the cancer screening campaigns and late presentation to the healthcare providers at incurable stages of cancer have been reported [16]. Early detection, seeking proven treatment at curable stages and patients' adherence to the cancer treatments are some of the important steps in prolonging the length and quality of life of cancer patients as well as reducing the cancer burden on the health care system. Malaysia is a country of multiethnic populations with different cultural and educational backgrounds, thus the evaluation of knowledge and perceptions of cancer and its treatment will be both fascinating and time consuming but necessary for a greater understanding of the causes for the late presentations.

The current study aims to explore cancer patients' experiences and knowledge about cancer and to explore the perceptions towards conventional cancer therapies.

## Methods

### Design and setting

The study was approved by the Medical Research Ethics Committee, Ministry of Health, Malaysia. Because little research has been undertaken in Malaysia to identify cancer patients' perceptions towards cancer and experiences with cancer treatments, qualitative research was adopted to explore the issue [17-19]. The study was conducted at Penang General Hospital, which provides major oncology services not only for the state of Penang but also for the neighbouring states including Kedah, Perlis and Perak. The hospital is capable of providing all major diagnostic and surgical procedures for cancer patients, and radiotherapy is given to the patients in a private hospital (Mount Miriam Cancer Hospital). The oncology department is comprised of an oncology ward, palliative care unit and day-care unit, which provide services and treatments to approximately 1000 cancer patients per year.

### Participants

The participants were selected from the three major ethnic groups in Malaysia, namely Malay, Chinese and Indians from February until July 2010. Patients 18 years of age or older, diagnosed with cancer at least six months ago and not more than five years post diagnosis that were on active cancer treatment at the Oncology wards of Penang General Hospital were approached. The principal investigator recruited patients for participation in this study. Informed consent was obtained from each respondent by giving the patient an information sheet in Malay and English to be read prior to the interviews. Patients who were unable to read or understand these two languages were given verbal information by the research assistant in their native languages. The interviews were continued until the saturation point was reached, when no new information was obtained from subsequent interviews. A total of 20 consenting cancer patients were interviewed.

### Study Tool

A semi-structured interview guide was used as a study tool "see Additional file 1". The interview guide was developed after extensive literature review. A list of possible questions to assess the patients' knowledge of cancer, treatment-seeking behaviour and treatment options were identified from the literature. While creating the questions, the focus was to keep the questions as open as possible to give interviewees maximum opportunity to express their views. The first draft of the interview guide was discussed among the authors and was modified after a few rounds of discussion. Pre-testing of the interview guide included conducting pilot interviews with five cancer patients to check whether particular questions were useful in the retrieval of information [20]. Specific probes were identified during the pilot interviews and the interview guide was subsequently modified.

### Procedure and Interview process

Interviews were conducted in the Malay language; however, Tamil and English languages were also used for patients who prefer to communicate in these two languages. We appointed two research assistants form Indian and Malay ethnic backgrounds to help in the interview process. Chinese patients were interviewed either in Malay or English. The interviews lasted for approximately 60-90 minutes. All interviews were audio-recorded so that verbatim transcriptions could be created. The principal investigator attended all interviews with the research assistant to take field notes and facilitate the interview process. Each interview was transcribed verbatim and was sent to the participants for approval. A minimum of one week was given to the patient to approve the transcript by signing the validation form. Patients' demographic-, disease- and treatment-related data were obtained from a questionnaire attached to the patient information sheet and the consent form. The interviews mainly focused on cancer patients' beliefs about cancer, its causes and perceptions of modern cancer treatment. Furthermore, information was gathered regarding their previous experiences with cancer and its possible effects on their treatment-seeking behaviour. Probing questions were used where necessary to get a more thorough understanding of the issue. Transcribed interviews were subjected to thematic content analysis, and the transcripts were analysed for relevant content to identify the emerging categories[21].

## Results

Twenty cancer patients (P1-P20) aged between 18 and 70 years (mean = 53years) were interviewed. Because Malays are the most populous ethnic group in Malaysia, they accounted for the highest (n = 10) number of patients in the sample group, followed by Indians (n = 6) and Chinese (n = 4). The majority of the participants were from a low-income group seeking treatment in government hospitals. All of the patients except one were not medically insured; therefore, government hospitals were the only choice for them to seek treatment. Patients' demographics and disease-related data are summarised in Table 1.

**Table 1:** Demographic Characteristics of the Cancer Patients Interviewed

Characteristics	n	%
**Age range**		
18-30	1	5
31-40	5	25
41-50	5	25
51-60	6	30
61-70	3	15
		
**Gender**		
Male	7	35
Female	13	65
		
**Race or Ethnicity**		
Malay	10	50
Indians	6	30
Chinese	4	20
		
**Education**		
Primary	8	40
Secondary	8	40
Matriculation/Diploma	4	20
		
**Socioeconomic status**		
Low (Less than RM* 1000/month)	10	50
Middle (RM 1000-RM 3000/month)	4	20
High (RM 3100 and above/month)	6	30
		
**Tumor Site**		
Naso-pharynx	3	15
Colorectal	5	25
Breast	5	25
Cervix	5	25
Liver	1	5
Non-Hodgkin's Lymphoma	1	5
		
**Tumor Stage**		
Stage 0 (In situ)	1	5
Stage I (Localized to one part)	3	15
Stage II & III (Locally advanced)	8	40
Stage IV (Metastasized to other organs)	8	40

During the analysis, three categories were identified: beliefs of the causes of cancer, self-belief of the effectiveness of conventional therapies for cancer treatment and personal self-belief and experiences regarding conventional medicines' adverse effects.

### Beliefs regarding the causes of cancer

When asked about why people get cancer and the reasons for getting cancers, cancer patients gave different reasons according to the following sub themes: unknown reasons, internal and external factors and spiritual attributions.

#### Unknown Reasons

In this preliminary investigation, cancer patients described cancer as a disease present in every human being that may become visible during the course of life.

*"Initially I thought cancer is a kind of sickness, that only fat people can get, but now when I came to the wards I noticed even thin people also get cancer. What I believe is everyone got cancer but it is not explored yet" *[P1-Nasopharyngeal carcinoma].

Patients reported cancer as a disease, which has no geographical or racial boundaries.

*"Anybody can get cancer. There is no way that you can control cancer, irrespective of age, race, diet, exercise... anyone can be a suitable candidate of cancer, except you have some ways to stop the normal cells to be converted into a cancerous cell*"[P12-Colon cancer].

#### Internal factors

Haematological imbalances and genetic predispositions were some of the internal factors reported by the cancer patients. Patients with a family history of cancer strongly believed cancer to be a genetic disease.

*"It happens due to the imbalance of the blood cells*"[ P6-Colon cancer].

*"Cancer is related to family history........ right? My mother passed away due to stomach cancer. Cancer doesn't discriminate. I have inherited the cancer from my mother" *[P16- Cervical cancer].

For those with a family history of cancer, additional inquiries were made to evaluate the perceived susceptibility to cancer.

*"Yes, my father at the age of 63, and my younger sister at the age of 20 years died of stomach cancer, but I never thought of getting cancer, because I was healthy all the time*" [P6-Colon cancer].

#### External factors

Unhealthy lifestyle habits such as smoking, lack of exercise and the presence of pesticides in food were some of the environmental factors believed to contribute to cancer.

*"You know the food you take may be you will get the cancer, because now the food you know, they put what? (what to say ... supplements... no no), ya... pesticides they put on the vegetables, I think that is the main cause of cancer'' *[P9-Colon cancer].

*"I was a heavy smoker and I use to smoke 20 cigarettes per day. I think my smoking habit must be one of the reasons I got cancer*" [P3-Colon Cancer].

Interestingly, some of the colon cancer patients related cancer to their eating habits containing such ingredients as spicy food and red meat.

*"May be if we eat spicy food the seeds of chilli may stuck in the stomach and may cause cancer" *[P3-Colon Cancer].

However, patients with eating habits that consisted of less spicy food and who also claimed to have a healthy lifestyle, such as regular exercise or a vegetarian diet, rejected the idea of an unhealthy lifestyle as a cause of cancer.

*"I don't think so, if that is the case, I think Malay and Indians should get more colon cancer, because they eat more spicy food than Chinese'' *[P14-Colon cancer].

*"To me, defining healthy and unhealthy lifestyle is a big problem, what do you mean by healthy lifestyle? A person who never did exercise, just have a normal daily routine work and then sleeping can stay very long also. Diet also what I eat my wife also eat the same, but I got (cancer) she did not" *[P12-Colon cancer].

#### Spiritual attribution

Although the majority of the patients gave different reasons and theories to the cause of their cancer, a strong spiritual connection was found as all patients described their cancer diagnosis as *God's will*.

*"Everyone will have to face it (death) regardless of where they are. No one can escape. That's actually our luck. If it has stated we ended our life with this kind of disease, we need to accept the destiny" *[P17-Cervical cancer].

In addition, spirituality was mentioned as one of the ways to control the emotions that can assist in boosting the immune system and increasing their chances of a cure.

*"Cancer is a mental disease rather than a physical. Spiritually, if you can control your emotions, your survivor rate is good. It is just when you are down emotionally, then it will affect your immune system, it will drop" *[P12-Colon cancer].

It was found that patients tend to be more enthusiastic in learning about their disease and trying to find probable reasons for having the disease, particularly relating it to their lifestyle habits and family history. However, it is also noted that patients, despite having a strong family history of cancer, never considered cancer screening prior to their diagnosis. Most of the patients showed a willingness to ask their close family members to go for regular health screenings. Despite differing expectations on their possible cure almost all patients reported spiritual practices such as praying and physical exercises, including dancing and yoga, as coping strategies for their ailment.

### Self-belief regarding the effectiveness of conventional therapies for cancer treatment

#### Positive attitude regarding conventional therapies

##### Scientifically proven

Participants expressed a variety of perspectives regarding the effectiveness of conventional therapies for cancer treatment. When asked about the quality of treatment from hospitals, the majority of the patients found it useful. They believed that their cancers could be cured from these therapies. Advancements in new technologies regarding effective treatment of cancer were the reasons given by the patients. Patients reported modern therapies were different from the traditional ones where the methods of preparations and doses have been the same for centuries.

*"Hospital medicines are the best, because I believe it is scientifically proven and the research is on all the time. I believe they (modern medicines) ensures complete cure" *[P6-Colon cancer].

##### Fixed dosing system

Fixed dosing system was given as one of the reasons patients perceived modern therapies as effective. Patients compared it with the traditional therapies where the medicines are usually given without any fixed dose or method of administration. Study participants also reported cancer as a disease that needs complete eradication of cancer cells, and a proper regimen after regular intervals is important, which they perceived as deficient in traditional ways of healing.

*"Yes, for me chemotherapy is the best way to treat cancer. Because medicines given by the doctors have certain dose, we know what will be the side effects and how to deal with them, not like traditional medicines, no specific dose at all, we are not sure what side effects, will we suffer and how bad are they to our body" *[P4-Breast cancer].

##### Treatment cost

Interestingly, high treatment cost was given as one of the reasons patients perceived modern therapies to be effective. Because traditional medicines can be bought easily from the local markets at cheap prices, some patients perceived them as ineffective for the treatment of cancer.

*"Like these hospital medicines you know it is very expensive, you cannot afford sometimes and it's not as easy to buy like traditional medicines, if you check the price per cycle... is around few thousand Malaysian Ringgit (RM)....so there must be some cure that is why they are so expensive....right?*" [P14-Colon cancer].

#### Negative attitude regarding conventional therapies

The study finding also shows that patients at the metastasis stage had a slightly negative perception of the effectiveness of modern therapies. Still, these patients were compliant with the therapies recommended by their oncologists, but due to the poor prognosis, they viewed modern therapies as only a way to prolong their life span.

*"No I don't think so, cancers are curable, the therapies given can just delay the process of death....the process of treatment is actually delaying the final outcome (death)" *[P12-Colon cancer].

*"I think we just want to prevent it from getting worst or to prolong the life only. Because you know cancer runs through blood and it can reach to other places very fast, so just to control that we need to take these therapies" *[P14-Colon cancer].

Because the study participants were getting treatments from the hospitals, it is possible that they already have a positive perception regarding conventional therapies. However, this study findings can help into identify the basis of their perceptions of the effectiveness of conventional therapies. Some participants compared the effectiveness of conventional therapies with the traditional ones and many described the lack of safety profile and poor efficacy of traditional medicines as reasons for opting for conventional treatment. There was however, some negative perception regarding the effectiveness of conventional therapies among patients at advanced stages and this demands an in-depth investigation to ensure patients' compliance with palliative care management.

### Personal self-belief and experiences regarding conventional medicines' adverse effects

#### Fear of side effects due to conventional therapies

Most of the participants reported a fear of side effects from chemotherapy and radiotherapy. Other major worries include the chances of survival at the time of cancer diagnosis. Despite the latest developments in cancer care and the numerous ways to avoid the side effects due to chemotherapy and radiotherapy, a strong negative perception was observed among cancer patients, which accounted for defaulting on proven therapies in some cases.

*"After surgery doctors advised me to go for chemotherapy, but I refused, I felt I am not strong for chemo, as I heard drugs for cancer are very hot" *[P10-Breast cancer].

Lack of knowledge regarding currently available conventional therapies and their side effects, as well as beliefs that are strongly influenced by the previous experiences of family or friends were among the reasons strengthening the fear regarding conventional therapies.

*"But to hear that chemo is the therapy, my tears went down automatically. We are not familiar with the chemo and somehow others tell us that the chemo is terrible and exhausting and causes too many side effects. I'm not afraid with the hair lost but I fear the pain*" [P19-Cervical cancer].

*"I've seen a relative who defecates in bags. He also did chemo for his intestinal cancer. If I'm not mistaken, he couldn't afford to finish his third chemo and died. He was already old (70 years old) and chemo made him lethargic. He died before getting his third cycle of chemo" *[P17-Cervical cancer].

#### Fear of surgery

Fear of surgery and removal of cancerous organs were some of the reasons given by patients to explain the delay in seeking conventional therapies.

*"Some people told me that after removing the organ, there would be other risks because as aging occurs, we will not be fit as before. The effects (of aging) will be faster*" [P17-Cervical cancer].

*"My family and relative stopped me not to see the doctor. They said, if once the body touched by knife or sharp object (referring to the surgery) it will make my health condition worst" *[P10-Breast cancer].

#### Positive attitude regarding the side effects due to conventional therapies

Although there had been some initial misconceptions and fear of side effects of conventional therapies, the majority of the cancer survivors showed a positive attitude in coping with the general side effects after chemo or radiotherapies.

*"Yes I lost my hair, I was bald but after that it grown black, beautiful black hair, I always tell this to my friends (those having cancer), if you want black hair you should go for your chemo. I never skipped my treatments and appointments" *[P2- Breast cancer].

Another cancer survivor reported that to accept the side effects as a reality was important, in contrast to denying these effects, a path that would just lead to treatment failure and nothing else.

*"You have to accept that there are good and bad effects of all drugs...true or not? You see my life for the past 5 years, I come to the ward for chemotherapy for 3 days and 2 nights, than later on, it takes 3 days to recover from the side effects, so my life is 1 week of chemo and than another week with the side effects and the third week I will come back for my chemo again. So you just learn to accept the side effects, there is no such thing as 'No side effects'. When you eat rice too much also you will get diabetes, if you take too much of salt also you will get high blood pressure*" [P12-Colon cancer].

Fear of chemotherapies appears to be the main factor that drives patients away from the proven therapies and may push them towards seeking unproven therapies. However, the perceptions appeared to change as patients continue with the modern therapies, and effective measures had been taken to reduce the side effects as much as possible, coupled with proper counselling and social support.

## Discussion

The objective of this study was to explore cancer patients' perspectives regarding cancer, as well as to understand their experiences relevant to modern cancer therapies. The current study portrays the participants from a local hospital in Malaysia, and represents a diverse group in terms of their age, ethnic background, cancer type and stage. As reported previously [22,23], risk perceptions of cancer are deeply influenced by specific cultural beliefs, we hypothesised that there will be differences in beliefs regarding the causes of cancer among patients from different ethnic backgrounds; this appeared not to be so, as patients related it more to their lifestyle habits and family history rather than any specific cultural beliefs. Genetic risk factors and their potential influence on the process of risk perception have been widely evaluated through qualitative investigations [24]. In a review of qualitative studies regarding ways in which lay people construct and experience cancer risk, it is concluded that risk perception is strongly influenced by one's own experience of having or caring for cancer patients [25]. Despite having a strong family history of cancer, the risk perceptions among the study participants were found to be very low, and a sense of perceived susceptibility to cancer was completely absent. The concept of perceived susceptibility has been found to be predictive of engaging in protective health behaviours [26]. During the discussion, most of the participants did not admit to having cancer screening prior to their diagnosis. In general, the likelihood of engaging in screening activities for cancer depends only on how much patients found themselves vulnerable of getting cancer. Our study provides basic information to clinicians regarding the importance of incorporating health beliefs prior to designing any educational or interventional programs.

The life style habits such as smoking, is no longer in debate as a potential cause of cancer. In the US, smoking accounts for at least 30% of all cancer deaths and 87% of lung cancer deaths [27]. It is distressing to note that despite numerous efforts aimed at discouraging smoking among Malaysians, every day approximately 50 teenagers under the age of 18 begin smoking [28]. A focus group investigation among smokers in Malaysia concluded that misconceptions and false beliefs such as 'smoking after food' or 'drinking water after smoking to moisturise the throat or lungs' to prevent harmful effects of smoking clearly indicate a poor level of knowledge about smoking and its harmful effects on health [29]. A sign of clear disappointment and self-guilt observed during our interviews where patients admitted their smoking habits as a reason for their cancer. An in-depth investigation about lay beliefs should be conducted and must be incorporated prior to designing any health campaigns. Study participants with good dietary and lifestyle habits rejected the idea of lifestyle habits as a cause of cancer. Patients related it more to luck or God's will. Despite being from three different religions, i.e., Islam, Hinduism and Buddhism, patients repeatedly used the word God, and God's will when referencing being diagnosed or possibly being cured of cancer. Many acknowledged that the diagnosis of cancer is a wakeup call to absolve oneself from sin or other spiritual contamination. Consistent with our findings, spirituality has been found to be one of the widespread strategies among cancer patients coping with cancer [30-32]. The participants of the study described numerous methods of coping, such as reciting verses from the Holy Book '*Al Quran' *among Muslims, regular visits to the temples among Hindus and numerous types of faith healing physical exercises among Buddhist cancer patients.

The study results suggest that the perception of a treatment plays an important role in treatment decision-making. Patients demonstrated an acceptable awareness and knowledge regarding modern therapies. The most dominant reason why patients perceived the conventional modern therapies as effective was the scientific method of preparation. At the same time, the perceived poor effectiveness of traditional therapies in curing cancer describes the importance of patients' knowledge about effective ways to treat cancer. During the last few decades, most of the studies focused on the reasons why patients used traditional medicines; however, patients in this study provide the evidence as to why they decided to seek modern therapies [33,34]. During the discussion, numerous themes did emerge about the perceived effectiveness of traditional medicines for cancer cure, but as this is not the part of the objectives of this study, this will not be discussed. It is imperative to note that Malaysians are reported to spend approximately $500 (US) million on traditional medicines, compared with only $300 (US) million for western medicines; however, little is known about how they perceived the effectiveness of these modalities, their potential risks and the duration of use [35]. Further analyses of the interviews of the participants could throw some light onto these perceptions. The Malaysian government has taken a proactive step in implementing the integrative medicines in some of the government hospitals. The desire of the government is to encourage the rational use of traditional medicines by registered traditional practitioners for chronic disease patients including cancer; however, patients' and physicians' perceptions regarding such integration are still questionable [36].

Cost of treatment is reported as a barrier in seeking treatment among cancer patients [37]. However, our results showed that the participants perceived modern cancer treatments as effective due to the high cost; additionally, the high cost was a reason for compliance to their treatment. The Malaysian health care system offers free treatment to all Malaysians for most of the chronic diseases; nevertheless the high cost of modern therapies sometimes forces the public to fork over their life savings.

The adverse effects of conventional treatments and its psychological effects on patients' quality of life have been widely studied among cancer patients [38-40]. In some cases, perceived severity of side effects due to conventional therapies is one of the reasons for partially or completely defaulting proven therapies [41]. Our findings were consistent with others, where fears of undesirable effects of surgery on the body and side effects due to chemotherapies were a few of the initial reasons in delays in seeking cancer treatment. At the same time, effective management of the side effects was also found to support patients' preference to continue with modern therapies. It should be appreciated that innovative methods such as effective anti-emetic regimen with newer agents and fully implantable venous devices are some of the initiatives taken by the government to improve cancer patients' compliance towards modern cancer therapies [13].

### Limitations

The study participants were receiving treatment in the hospitals at the time of interview, which may explain some of their positive attitudes towards conventional therapies. Conducting similar studies among patients who defaulted their therapies partially or completely may give better insights into the perceived effectiveness of conventional therapies to cure cancer. The paucity of the funding restricted the study to only one hospital in Malaysia; however, significant efforts were made to include as wide a range of patients from diverse ethnic backgrounds and a variety of different types of cancer which may help to generalize the data to some extent.

## Conclusions

This qualitative exploratory study investigated the beliefs and experiences of cancer patients with cancer and its treatment. Spiritual attribution regarding the causes of cancer is found to be an effective way to cope with the psychological distress faced by most of the cancer patients. In recognising patient's beliefs at the time of treatment decisions, patients' compliance with proven therapies can be improved and may help in removing myths about cancer treatment. The study finding also provide ground information about the cancer patients knowledge of cancer and perceived effectiveness of modern treatments and can be helpful to design more cultural specific educational program. This in turn, may improve awareness regarding cancer screening and the importance of seeking treatment at early stages.

## Authors' details

^1^Faculty of Pharmacy, Universiti Teknologi MARA (UiTM), Pulau Pinang, Malaysia, ^2^Discipline of Social & Administrative Pharmacy, Universiti Sains Malaysia (USM), Pulau Pinang, Malaysia, ^3^Advanced Medical and Dental Institute, Universiti Sains Malaysia (USM), Pulau Pinang, Malaysia, ^4^Department of Radiotherapy and Oncology, General Hospital Penang, Pulau Pinang, Malaysia, ^5^Department of Medicine, Allianze University College of Medical Sciences (AUCMS), Pulau Pinang, Malaysia

## Competing interests

The authors declare that they have no competing interests.

## Authors' contributions

MF conducted the filed work and drafted the manuscript. MAH, AKS and AAS conceived and supervised the project. TBS and MAF helped in analysis and manuscript review. All authors read and approved the final manuscript.

## Pre-publication history

The pre-publication history for this paper can be accessed here:

http://www.biomedcentral.com/1471-2458/11/525/prepub

## Supplementary Material

Additional file 1 **Interview guide**. File providing the details of the questions designed and used as a study tool during the interviews. Click here for file
