# Supplementary material for: A qualitative exploration of Malaysian cancer patients' perspectives on cancer and its treatment
Source: BMC Public Health. 2011 Jul 1;11:525. doi: 10.1186/1471-2458-11-525 (PMC3146868; doi:10.1186/1471-2458-11-525)
Supplement: Additional file 1 — Interview guide. File providing the details of the questions designed and used as a study tool during the interviews. [file 1471-2458-11-525-S1.DOC]

### Additional file 1 – Interview guide

**Questions to assess Knowledge about cancer**

1. Do you know your cancer type and the treatment you have received so far?
2. Can you tell me what cancer is? How does it happen? Who can get it? Why?
3. Do you think cancer is related to our health style?
4. Do you think cancer is a curable disease?

**Questions to assess the treatment-seeking behaviour**

1. How long did it take you to seek treatment after the diagnosis of cancer?
2. Were there any delays in seeking treatment? If yes, what were the reasons? (Specific probes: work or household responsibilities/lack of time, influence of the eastern medicines, lack of knowledge about cancer treatment, fear of side effects due to conventional therapies, lack of health care facilities)

**Questions to assess the cancer treatment options**

1. What is the best option to treat cancer? (Specific probe: Modern therapies available at the hospitals or traditional therapies)
2. Do you think conventional therapies are effective and working on you?
3. Do you know the importance of completing chemotherapy cycles?
4. Were there any side effects due to conventional therapies?
